# Fraternal twins with Phelan-McDermid syndrome not involving the *SHANK3* gene: case report and literature review

**DOI:** 10.1186/s12920-020-00802-0

**Published:** 2020-10-06

**Authors:** Shan Li, Ke-wang Xi, Ting Liu, Ying Zhang, Meng Zhang, Li-dong Zeng, Juan Li

**Affiliations:** 1 The First School of Clinical Medicine, Lanzhou University, Lanzhou, China; 2 Central Laboratory, The First Hospital of Lanzhou University, Lanzhou, China; 3 GeneMind Biosciences Company Limited, Shenzhen, China

**Keywords:** Phelan-McDermid syndrome, *SHANK3*, 22q13 interstitial deletion, Neurodevelopmental disorders

## Abstract

**Background:**

Phelan-McDermid syndrome (PMS, OMIM#606232), or 22q13 deletion syndrome, is a rare genetic disorder caused by deletion of the distal long arm of chromosome 22 with a variety of clinical features that display considerably heterogeneous degrees of severity. The *SHANK3* gene is understood to be the critical gene for the neurological features of this syndrome.

**Case presentation:**

We describe one pair of boy-girl twins with a 22q13 deletion not involving the *SHANK3* gene. Interestingly, the clinical and molecular findings of the two patients were identical, likely resulting from germline mosaicism in a parent. The boy-girl twins showed intellectual disability, speech absence, facial dysmorphism, cyanosis, large fleshy hands and feet, dysplastic fingernails and abnormal behaviors, and third-generation sequencing showed an identical de novo interstitial deletion of 6.0 Mb in the 22q13.31-q13.33 region.

**Conclusions:**

Our case suggests that prenatal diagnosis is essential for normal parents with affected children due to the theoretical possibility of parental germline mosaicism. Our results also indicated that other genes located in the 22q13 region may have a role in explaining symptoms in individuals with PMS. In particular, we propose that four candidate genes, *CELSR1*, *ATXN10*, *FBLN1* and *WNT7B*, may also be involved in the etiology of the clinical features of PMS. However, more studies of smaller interstitial deletions with 22q13 are needed to corroborate our hypothesis and better define the genotype-phenotype correlation. Our findings contribute to a more comprehensive understanding of PMS.

## Background

Phelan-McDermid syndrome (PMS, OMIM#606232), also referred to as 22q13 deletion syndrome, is a rare genetic disorder caused by deletion of the distal long arm of chromosome 22 with a variety of clinical features that display considerably heterogeneous degrees of severity. This syndrome is characterized by global developmental delay, intellectual disability, absent or severely delayed speech, hypotonia, minor dysmorphic features and autism spectrum disorder (ASD) [1]. The *SHANK3* gene has been identified as the critical candidate gene for the neurological features of this syndrome [2]. However, previous genotype-phenotype studies of PMS [3–7] and several case reports of 22q13 interstitial deletion [8–10] have implied the role of additional genes or regulatory regions proximal to the *SHANK3* gene in PMS. Here, we report identical clinical and molecular findings from one pair of boy-girl twins with a de novo interstitial 22q13.31-q13.33 deletion not involving the *SHANK3* gene. To the best of our knowledge, this is the first report of fraternal twins with PMS not involving the *SHANK3* gene.

## Case presentation

Our patients are seven-year-old boy-girl twins who were born from the first pregnancy of healthy unrelated parents (a 32-year-old mother and a 37-year-old father) of Chinese descent with unremarkable family histories. The patients were conceived by in vitro fertilization because their mothers’ fallopian tubes were blocked. No abnormality was identified on prenatal ultrasonography. The serum triple-marker (including alpha-fetoprotein, free-β-human chorionic gonadotropin, and unconjugated estriol) screening test result for Down syndrome was negative. The twins were born at 40 weeks gestation by caesarean section with some minor complications. At birth, the foreheads, cheekbones, wrists and ankles of the two infants were cyanotic, and they both cried weakly; the condition of the boy was more serious than the condition of the girl. To date, the cyanosis has not disappeared but is better that observed at birth. The degree of jaundice was also more serious in the boy than in the girl. As a result, the boy was not discharged until 7 days after bili light treatment. The boy was born with thyroid cartilage hypoplasia that spontaneously resolved after 1 month, whereas the girl did not exhibit this complication.

Both infants showed infantile hypotonia and feeding difficulties and could not control their heads until they were 2 years old. At the age of 6 months, their parents took them to the child health department for routine medical examinations. The twins underwent a neuropsychological development examination, which was carried out using the Chinese version of Gesell Development Scale (GDS). Infant development was assessed using the development quotient (DQ) according to the following criteria: normal (DQ ≥ 85), borderline (75 ≤ DQ < 85) and abnormal (DQ < 75). The girl had DQ of 60 for “gross motor”, 55 for “fine motor”, 35 for “language”, 47 for “adaptive behavior” and 48 for “personal-social behavior”. The boy had DQ of 58 for “gross motor”, 54 for “fine motor”, 32 for “language”, 45 for “adaptive behavior” and 46 for “personal-social behavior”. The results showed that they had intellectual disability. Subsequently, the clinician advised them to have trace element (including calcium, magnesium, iron, copper, zinc, lead and cadmium) and brain MRI examinations. Trace elements in the blood were normal, and brain MRI showed hypoplasia of white matter and external hydrocephalus. They were able to sit at 6 months and walk against a wall at 24 months, though their gait was not stable. At the age of 2–4 years, the twins underwent physical therapy; however, no significant clinical progress was observed. They then developed severe language disability and could not speak any words. They had abnormal social interactions, were no shy with poor eye contact and stereotypic behaviors and were interested in only one children’s song. These features indicated ASD in the patients, but they received no formal testing for autism. The girl and boy developed febrile convulsions at the age of 3 years and 4 years, respectively. When they experienced febrile convulsions, they were advised to have EEG (four-hour visual EEG, awake) and brain MRI examinations performed. The EEG and brain MRI results were normal, and the abnormalities observed in the first brain MRI had disappeared.

**Fig. 1:**
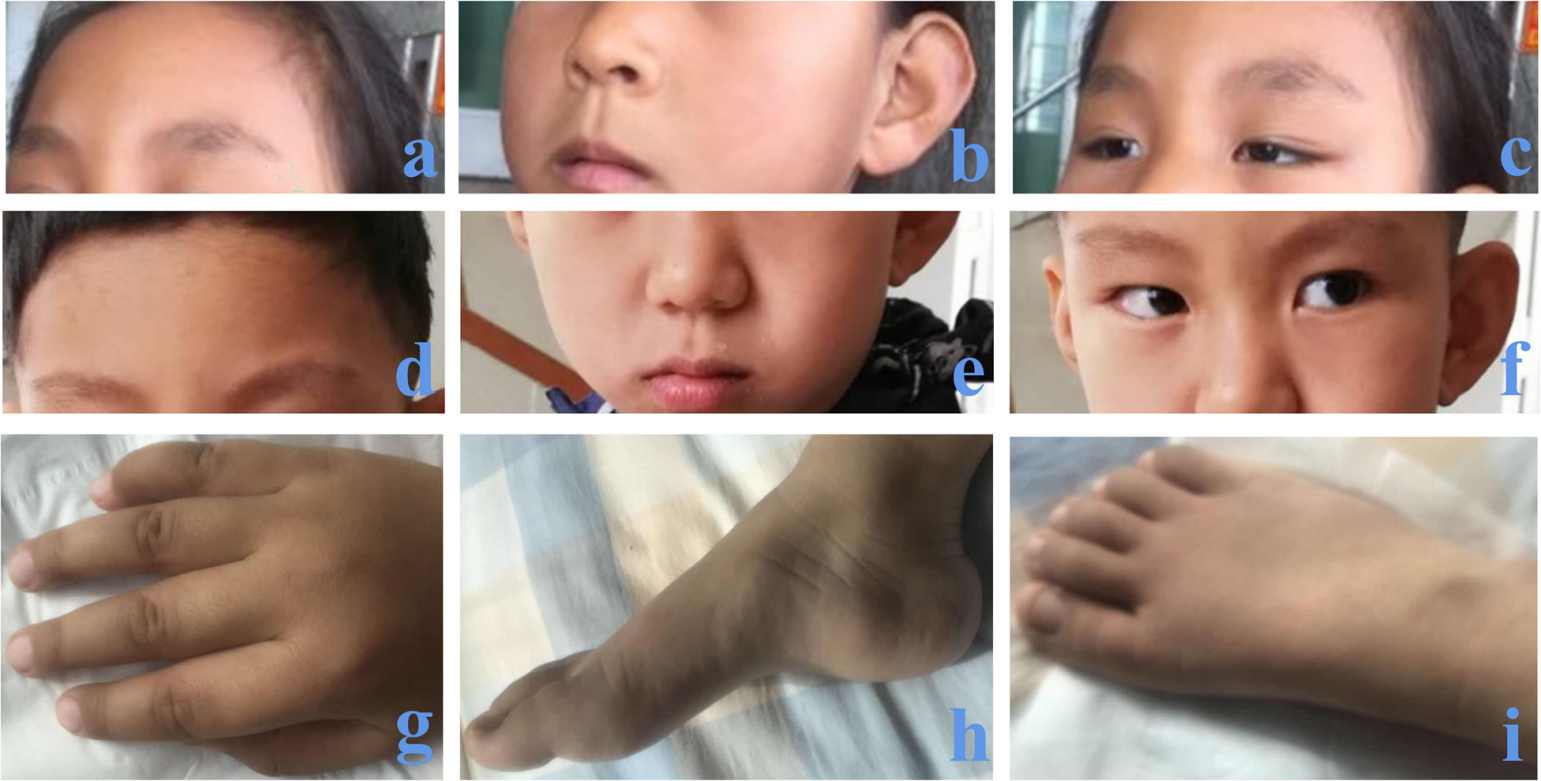
The girl at the ages of 5 (**a-c**) and 7 (**g-i**) years; the boy at the age of 5 (**d-f**) years. Both patients had dolichocephaly, large prominent ears (**b, f**), a prominent forehead (**a, d**), widely spaced eyes (**c, f**), bilateral ptosis (**c, f**), a bulbous nasal tip (**b, e**), a wide nasal bridge (**c, f**), a long philtrum (**b, e**), large fleshy hands and feet, fifth finger clinodactyly of the right hand and dysplastic nails (**g-i**, data of the boy not shown)

**Fig. 2:**
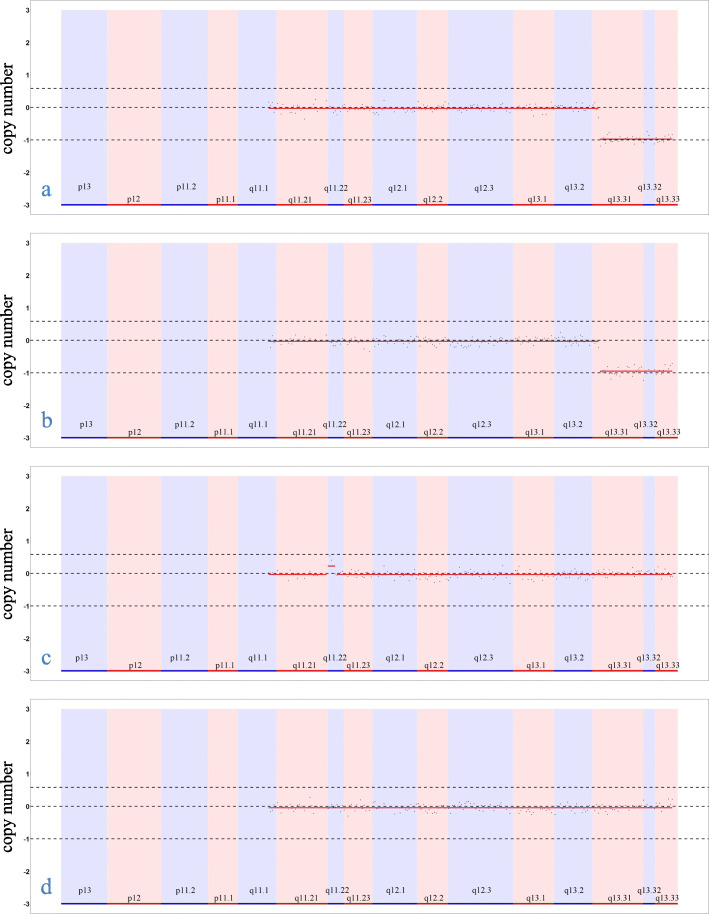
Third-generation sequencing results focusing on chromosome 22 in the probands’ family; 22q13.3 deletion is present in two probands (boy: **a**, girl: **b**) and absent in the parents (mother: **c**, father: **d**). The mother has a 0.6 Mb duplication on 22q11.22 (CNV ratio 35%)

At the age of 5 years, they walked without a sense of direction and showed some toe-walking behavior and a scissor-like gait. They were unable to travel up and down stairs independently, and their neck, wrist and ankle muscles were still weak. Mild facial dysmorphic features included dolichocephaly, large prominent ears, a prominent forehead, widely spaced eyes, bilateral ptosis, a bulbous nasal tip, a wide nasal bridge and a long philtrum. In addition, they showed large fleshy hands and feet, fifth finger clinodactyly of the right hand and dysplastic nails (Fig. 1). Initial genetic testing consisted of tandem mass spectrometry and karyotype analysis, the results of which were unremarkable. The karyotype analysis of the parents was also normal. Copy number variation sequencing (CNV-seq) analysis of the two patients revealed a heterozygous deletion involving the 22q13.31-22q13.33 region. The deletion sizes in the boy and the girl were 6.36 Mb and 6.34 Mb, respectively. To further confirm the size of the deletion, we reanalyzed the two patients and both parents by third-generation sequencing. DNA was isolated from peripheral blood samples obtained from the two patients and their parents using the Ezup Column Blood Genomic DNA Purification Kit (Sangon Biotech, Shanghai, China). Third-generation sequencing was performed on the single-molecule sequencer GenoCare at the GeneMind Biosciences Company Limited, Guangzhou, China. GenoCare sequencing was performed according to a previously disclosed protocol [11]. We found that the two patients’ deletions were identical; both were 6.0 Mb, with breakpoints at 44850001 bp and 50,850,001 bp (GRCh37/hg19). Carrier testing in the parents revealed normal results in the 22q13.31-q13.33 region, indicating a de novo 22q13.31-q13.33 deletion in their children (Fig. 2). The hemizygous region included 45 protein-coding genes not involving the *SHANK3* gene, 34 of which are OMIM genes (Additional file 1: Table S1).

**Fig. 3:**
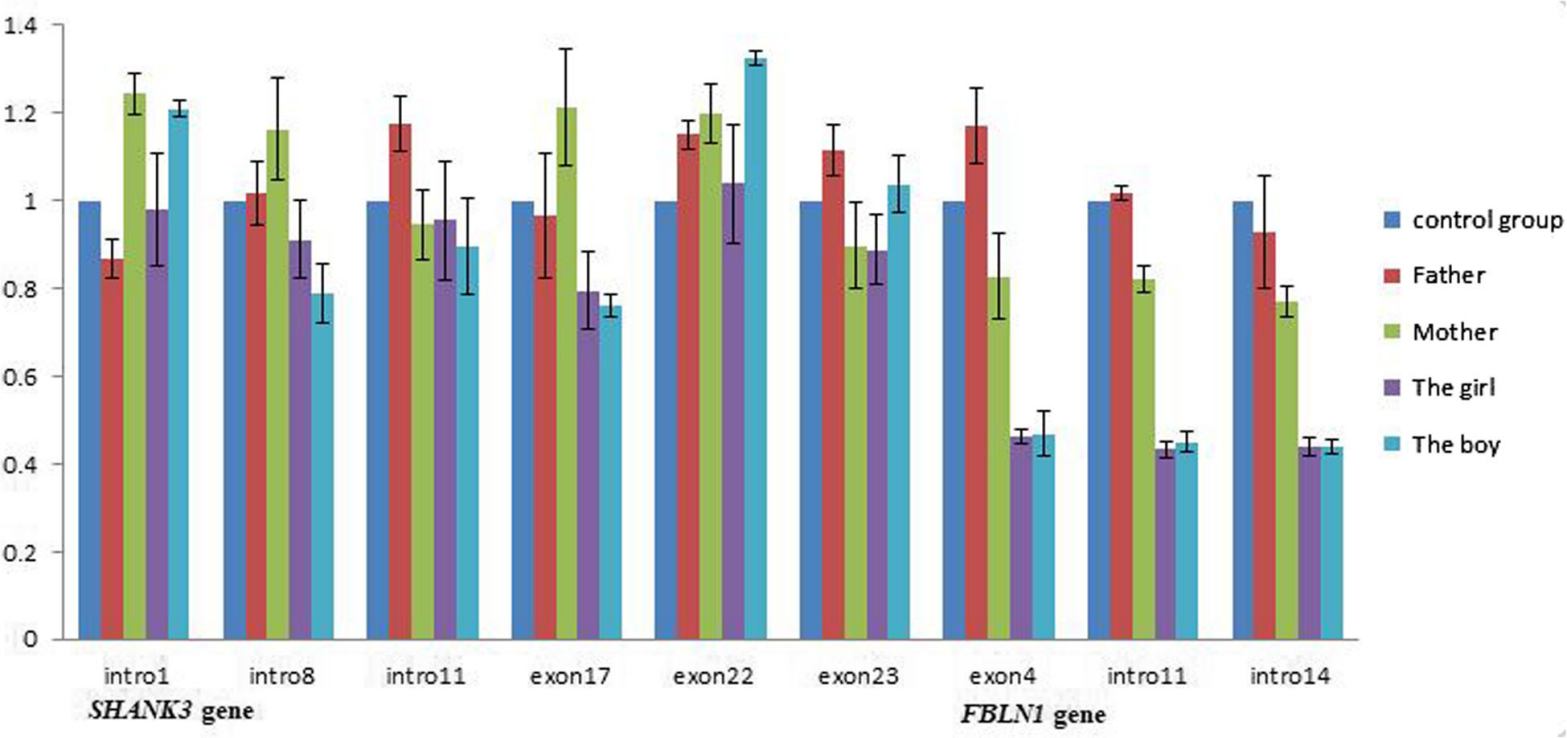
Quantitative PCR validation of the *SHANK3* gene and *FBLN1* gene (positive control). Relative quantitation (RQ) of the copy number was analyzed by the ^△△^CT method, and error bars represent the standard deviation. The *GAPDH* gene served as the reference gene (control group). Both copies of the *SHANK3* gene have been retained in the patients and their parents, and the copy numbers of the *FBLN1* gene in the patients were only approximately half of the values in the control group, confirming that the patients carry a de novo 22q13.31-q13.33 deletion not involving the *SHANK3* gene

Quantitative real-time PCR (qPCR) was conducted to validate the *SHANK3* gene and *FBLN1* gene (positive control) in the family. Genomic DNA was extracted from peripheral leukocytes using the QIAamp® DNA Blood Mini Kit (Qiagen, Germany). Partial exons and partial introns of the *SHANK3* gene and *FBLN1* gene were amplified using qPCR primers (Table 1). The *GAPDH* gene served as a reference gene (control group). Amplification and detection were performed on the LightCycler 480 II (Roche Diagnostics) using TB Green® Premix Ex Taq™ II (Takara, Japan). qPCR was conducted under the following cycling conditions: 95 °C for 30 s, followed by 40 cycles of 95 °C for 5 s and 60 °C for 30 s. Data were analyzed using the ^△△^CT method. The copy numbers of intro1, intro8, intro11, exon17, exon22, and exon23 of the *SHANK3* gene were approximately the same as the values in the control group, and the copy numbers of exon4, intro11, and intro14 of the *FBLN1* gene were only approximately half of the values in the control group, indicating that both copies of the *SHANK3* gene have been retained in the patients and confirming that the patients carry a de novo 22q13.31-q13.33 deletion not involving the *SHANK3* gene (Fig. 3).

**Table 1:** Quantitative real-time primers

No.	primer	position	Sequence(5′-3′)
1	*SHANK3*-1F	Intro 1	TGACCAGAGGCTGTTTTGAG
2	*SHANK3*-1R	Intro 1	CAGCAGATCCACCTCGACC
3	*SHANK3*-8F	Intro 8	CCTGCGCACGCCATGT
4	*SHANK3*-8R	Intro 8	GAGACCATCCGAGCACAACA
5	*SHANK3*-11F	Intro 11	CACCTGTGTAGTGATGGGCT
6	*SHANK3*-11R	Intro 11	CTCTCCACCTAACACGCTCC
7	*SHANK3*-17F	Exon 17	CGCCTCGTCATGAAGGTTGT
8	*SHANK3*-17R	Exon 17	CGAGCCCCGTCCTCTTCT
9	*SHANK3*-22F	Exon 22	GGAGAGCGGGGAACTCACT
10	*SHANK3*-22R	Exon 22	CTGTCCGAGGACTGCTTCAG
11	*SHANK3*-23F	Exon 23	ACTCATCCTTCCGCCAACAG
12	*SHANK3*-23R	Exon 23	CCCACAGGTGAGTGTGAGAC
13	*FBLN1*-4F	Exon 4	AGAGCTGCGAGTACAGCCT
14	*FBLN1*-4R	Exon 4	CGACATCCAAATCTCCGGTCT
15	*FBLN1*-11F	Intro11	GGACCTCTGTCTCTCCGAGT
16	*FBLN1*-11R	Intro11	ACCGCTCAGAGCATCATACG
17	*FBLN1*-14F	Intro14	TCCTCCCATGAGGGACTCAG
18	*FBLN1*-14R	Intro14	CACAGCCTTGGCCTGAAAAC
19	*GAPDH*-8F	Intro 8	ACTGGCTCTTAAAAAGTGCAGGGT
20	*GAPDH*-8R	Intro 8	TTGCTGTAGCCAAATTCGTTGTC

**Fig. 4:**
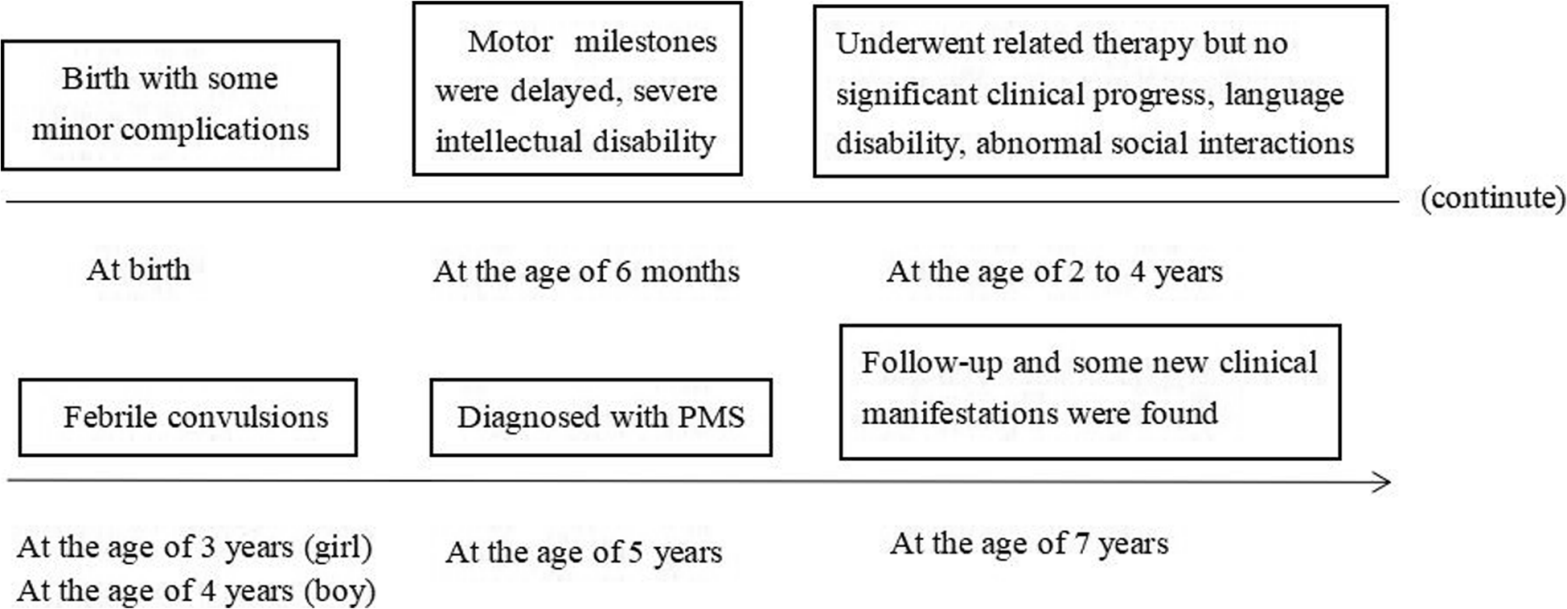
Medical history of the two patients

At the age of 7 years, The girl’s height was 120 cm (25-50th percentile), her weight was 25 kg (50-75th percentile), her body mass index was 17.4 kg/m^2^ (85th percentile) and her head circumference (HC) was 51 cm (74th percentile). The boy’s height was 115 cm (5-15th percentile), his weight was 23 kg (50th percentile), his body mass index was 17.4 kg/m^2^ (85-95th percentile) and his HC was 50 cm (22nd percentile). Both patients had increased pain tolerance and incontinence. The twins felt neither hungry nor satiety. They did not feed themselves or squat down, but they were able to move around obstacles. They did not know that any relatives or strangers could take them away. The boy had abnormal genitalia. The girl had sleep disturbances (easy to wake) and recurrent upper respiratory tract infections, while the boy did not. A timeline of historical and current information is shown in Fig. 4.

## Discussion and conclusions

In this study, we described identical clinical and molecular findings in one pair of boy-girl twins with intellectual disability, speech absence, facial dysmorphism, cyanosis, large fleshy hands and feet, dysplastic fingernails and abnormal behaviors. Using third-generation sequencing, we identified a 6.0 Mb de novo interstitial deletion of the 22q13.31-q13.33 region encompassing 45 protein-coding genes.

Several reports have indicated that although probands obviously have de novo deletions, siblings can have the same deletions, probably due to germline mosaicism in a parent, which is similar to our report [12, 13]. Germline mosaicism may be a significant mechanism for the generation of de novo pathogenic CNVs [14]. If the 22q13.3 variant found in the proband cannot be detected in the leukocyte DNA of either parent, the recurrence risk in siblings is estimated to be 1% because of the theoretical possibility of parental germline mosaicism, which is marginally greater than in the general population [15]. Therefore, prenatal diagnosis is essential for normal parents with affected children.

Previous genotype-phenotype studies have revealed that many clinical features of PMS are associated with deletion size. In a study of 201 patients, Sarasua et al. found that some neurologic and dysmorphic features, such as speech and developmental delay and macrocephaly, correlated with deletion size [3]. A genotype-phenotype study of 71 patients showed an association between increased deletion size and 16 features [4]. Samogy-Costa et al. found that renal abnormalities, lymphedema, and language impairment were positively associated with deletion sizes [5]. Moreover, 22q13.2q13.32 genomic regions were associated with the severity of speech delay, developmental delay, and physical features by statistical analysis in 70 patients with terminal 22q13 deletion [6]. Tabet et al. found that the absence of speech, ophthalmic features, and gastroesophageal reflux were associated with deletions of genome segments located at the 42.6–46.3 Mb region, 42.25–44.6 Mb region and 48.9–49.9 Mb region on chromosome 22, respectively [7]. Although the relationship between deletion size and phenotype has not been consistent across reports, the role of additional genes or regulatory regions proximal to the *SHANK3* gene has been suggested. Several case reports of 22q13 interstitial deletion support this view. The *SULT4A1* and *PARVB* genes have been suggested to be related to neurological features and macrocephaly/hypotonia, respectively [9]. In addition, Palumbo et al. proposed *CELSR1*, *ATXN10*, *FBLN1*, and *UPK3A* as candidate genes in the onset of the main clinical features of 22q13.31 microdeletion [10].

**Fig. 5:**
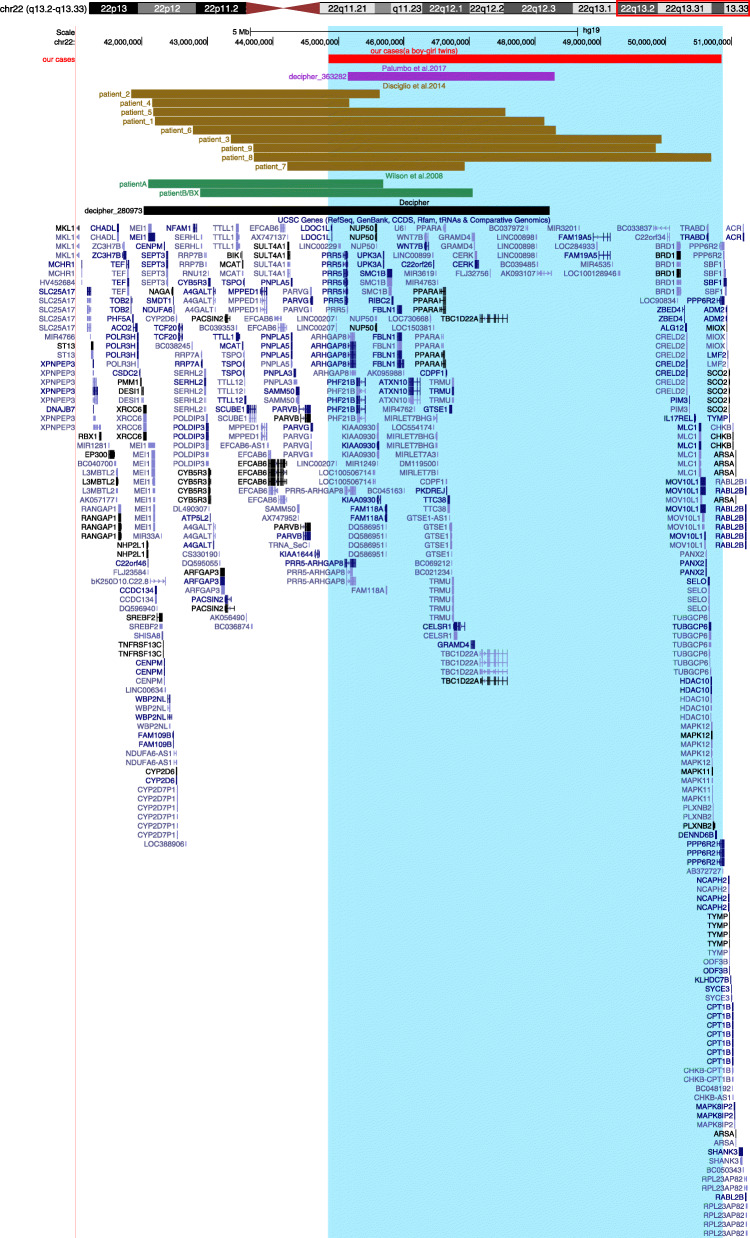
Mapping of the 22q13 deletions: our cases (red), one patient described by Palumbo et al. [2017] (purple), nine patients described by Disciglio et al. [2014] (brown), three patients described by Wilson et al. [2008] (green) and one patient from the DECIPHER database (black) in the UCSC Genome Browser (GRCh37/hg19). Our cases’ deletion region is represented by the blue-shadow box (not involving the *SHANK3* gene)

The phenotype of our patients described in the present study closely matches that of previously described patients with 22q13 interstitial deletions, namely, developmental delay/intellectual disability (DD/ID), speech delay/absence, hypotonia and malformations of the hands or feet. To further investigate genotype-phenotype correlations in 22q13 interstitial regions proximal to *SHANK3*, we collected 1 well-characterized patient from the DECIPHER database (https://decipher.sanger.ac.uk/) with good overlap with our patients. Additional file 2: Table S2 lists and compares the clinical and molecular findings of the probands and 14 previously reported cases, and a molecular comparison is shown in Fig. 5. Analysis of the data shows that consistent findings included DD/ID (15/16), delayed speech (15/16), hypotonia (12/16), macrocephaly (10/16), dolichocephaly (3/16), feeding problems (9/16), hands/feet anomalies (10/16) and facial dysmorphisms (14/16). Similar to all patients with a terminal 22q13 deletion, the 16 patients without a *SHANK3* deletion had DD/ID, delayed speech, hypotonia and dysmorphic features. Our findings further confirmed that other deleted genes in the 22q13 region likely contribute to the phenotypic characteristics of PMS.

The deletions identified in our two probands were identical and encompassed 45 protein-coding genes. Based on different analyses of the genes in the 22q13 region [2, 7, 16] and the pLI score (Additional file 1: Table S1) of these genes, we suggest that the genes *CELSR1*, *ATXN10* and *WNT7B* are also responsible for the neurodevelopmental clinical features observed in PMS patients, while *FBLN1* is a candidate gene that may explain hands/feet anomalies [17]. Our hypothesis is similar to that of Palumbo et al. [10]. The functions of the four candidate genes mentioned above are listed in Additional file 3: Document S1. The deletions in three previously described patients (patient 2, patient 4 and patient A) did not encompass the *CELSR1/ATXN10*/*WNT7B* genes, but their deletion region contained the *SULT4A1* gene, which may be associated with neurological symptoms in PMS [9]. To confirm our hypothesis, more patients, including those with only microdeletions of the *ATXN10*/*CELSR1*/WNT7B genes or point mutation carriers of these three genes, must be identified to determine whether their neurological development is affected. Thirteen of the 16 patients we described had a *FBLN1* gene deletion. Among them, 8/13 had hands/feet anomalies. The most common abnormalities were large fleshy hands and feet. Although not all patients with gene deletions show the phenotypes, the possibility that *FBLN1* may be related to hands/feet anomalies cannot be ruled out. Other mechanisms may affect the expression of the *FBLN1* gene, and larger case series and basic research are needed to confirm this hypothesis. No other PMS gene appears to be associated with hands/feet anomalies.

This study is the first report of one pair of boy-girl twins with PMS not involving the *SHANK3* gene. The clinical and molecular findings of the two patients were identical, likely resulting from germline mosaicism in a parent. Thus, we suggest that prenatal diagnosis is essential for normal parents with affected children. In addition, we found that the clinical phenotype of patients without *SHANK3* deletion was similar to that of patients with *SHANK3* deletion and further confirmed that other genes within this region may have a role in explaining symptoms in individuals with PMS. In particular, we propose that four candidate genes, *CELSR1*, *ATXN10*, *FBLN1* and *WNT7B*, may also be involved in the etiology of the clinical manifestations of PMS. However, more studies of smaller interstitial deletions with 22q13 are needed to corroborate our hypothesis and better define the genotype-phenotype correlation. Our findings contribute to a more comprehensive understanding of PMS.

## Supplementary information

**Additional file 1: Table S1**. Genes within the two probands’ deleted 22q13.31-22q13.33 segment and associated disorders, with the pLI score computed by ExAC (http://gnomad.broadinstitute.org/).

**Additional file 2: Table S2**. Clinical and molecular features of patients with overlapping 22q13 deletions not involving the *SHANK3* gene (the probands, DECIPHER patient and previously reported patients).

**Additional file 3: Document S1.** The functions of the four candidate genes.

## Data Availability

The datasets generated and/or analysed during the current study are available in the NCBI Sequence Read Archive (SRA) with the BioProject accession number. PRJNA664438 (https://www.ncbi.nlm.nih.gov/sra/?term=PRJNA664438). Additionally, database used in this study were DECIPHER (https://decipher.sanger.ac.uk/), the Exome Aggregation Consortium (ExAC) (http://gnomad.broadinstitute.org/) and human genome reference GRCh37/ hg19 (https://www.ncbi.nlm.nih.gov/assembly/GCF_000001405.13/).

## Abbreviations

PMS: Phelan-McDermid syndrome
ASD: Autism spectrum disorder
GDS: Gesell Development Scale
DQ: Development quotient
CNV-seq: Copy number variation sequencing
HC: Head circumference
DD: Developmental delay
ID: Intellectual disability

## References

[1] Phelan K, McDermid HE (2012). The 22q13.3 deletion syndrome (Phelan-McDermid syndrome). Mol Syndromol.

[2] Mitz AR, Philyaw TJ, Boccuto L, Shcheglovitov A, Sarasua SM, Kaufmann WE (2018). Identification of 22q13 genes most likely to contribute to Phelan McDermid syndrome. Eur J Hum Genet.

[3] Sarasua SM, Boccuto L, Sharp JL, Dwivedi A, Chen C-F, Rollins JD (2014). Clinical and genomic evaluation of 201 patients with Phelan–McDermid syndrome. Hum Genet.

[4] Sarasua SM, Dwivedi A, Boccuto L, Rollins JD, Chen CF, Rogers RC (2011). Association between deletion size and important phenotypes expands the genomic region of interest in Phelan-McDermid syndrome (22q13 deletion syndrome). J Med Genet.

[5] Samogy-Costa CI, Varella-Branco E, Monfardini F, Ferraz H, Fock RA, Barbosa RHA (2019). A Brazilian cohort of individuals with Phelan-McDermid syndrome: genotype-phenotype correlation and identification of an atypical case. J Neurodev Disord.

[6] Sarasua SM, Dwivedi A, Boccuto L, Chen C-F, Sharp JL, Rollins JD (2014). 22q13.2q13.32 genomic regions associated with severity of speech delay, developmental delay, and physical features in Phelan–McDermid syndrome. Genet Med.

[7] Tabet AC, Rolland T, Ducloy M, Lévy J, Buratti J, Mathieu A (2017). A framework to identify contributing genes in patients with Phelan-McDermid syndrome. NPJ Genom Med.

[8] Wilson HL, Crolla JA, Walker D, Artifoni L, Dallapiccola B, Takano T (2008). Interstitial 22q13 deletions: genes other than *SHANK3* have major effects on cognitive and language development. Eur J Hum Genet.

[9] Disciglio V, Lo Rizzo C, Mencarelli MA, Mucciolo M, Marozza A, Di Marco C (2014). Interstitial 22q13 deletions not involving *SHANK3* gene: a new contiguous gene syndrome. Am J Med Genet A.

[10] Palumbo P, Accadia M, Leone MP, Palladino T, Stallone R, Carella M (2018). Clinical and molecular characterization of an emerging chromosome 22q13.31 microdeletion syndrome. Am J Med Genet A.

[11] Zhao L, Deng L, Li G, Jin H, Cai J, Shang H (2017). Single molecule sequencing of the M13 virus genome without amplification. PLoS One.

[12] Tabolacci E, Zollino M, Lecce R, Sangiorgi E, Gurrieri F, Leuzzi V (2005). Two brothers with 22q13 deletion syndrome and features suggestive of the Clark-Baraitser syndrome. Clin Dysmorphol.

[13] Verhoeven WM, Egger JI, Willemsen MH, de Leijer GJ, Kleefstra T, treatment. Phelan-McDermid syndrome in two adult brothers: atypical bipolar disorder as its psychopathological phenotype? Neuropsychiatr Dis 2012; 8: 175. 10.2147/NDT.S30506 PMC3346055 22570549

[14] Moessner R, Marshall CR, Sutcliffe JS, Skaug J, Pinto D, Vincent J (2007). Contribution of *SHANK3* mutations to autism spectrum disorder. Am J Hum Genet.

[15] Rahbari R, Wuster A, Lindsay SJ, Hardwick RJ, Alexandrov LB, Turki SA (2016). Timing, rates and spectra of human germline mutation. Nat Genet.

[16] Ziats CA, Grosvenor LP, Sarasua SM, Thurm AE, Swedo SE, Mahfouz A (2019). Functional genomics analysis of Phelan-McDermid syndrome 22q13 region during human neurodevelopment. PLoS One.

[17] Debeer P, Schoenmakers EF, Twal WO, Argraves WS, De Smet L, Fryns JP (2002). The fibulin-1 gene (FBLN1) is disrupted in a t(12;22) associated with a complex type of synpolydactyly. J Med Genet.

