# Supplementary material for: Fraternal twins with Phelan-McDermid syndrome not involving the SHANK3 gene: case report and literature review
Source: BMC Med Genomics. 2020 Oct 6;13:146. doi: 10.1186/s12920-020-00802-0 (PMC7539423; doi:10.1186/s12920-020-00802-0)
Supplement: Supplementary file 3 — Additional file 3: Document S1. The functions of the four candidate genes. [file 12920_2020_802_MOESM3_ESM.docx]

Additional Document S1: The functions of the four candidate genes.

The *ATXN10* gene is thought to play an important role in the survival and differentiation of neurons or neuron-like cells [1].A heterozygous unstable tandem expansion of a pentanucleotide ATTCT repeat in the 9th intron of the *ATXN10* gene leads to spinocerebellar ataxia type 10 (SCA10, MIM 603516),an autosomal dominant neurodegenerative disorder characterized by cerebellar ataxia and seizures [2].To date, the underlying mechanism of SCA10 is not fully understood.Some studies show that loss of function of the *ATXN10* gene is unlikely to be the pathogenic mechanism of SCA10 [1, 3]. This view is supported by our work; that is, none of the 13/16 patients with *ATXN10* haploinsufficiency reported in Supplementary Table 1 showed clinical signs of SCA10. However, in view of the important role of *ATXN10* in neuronal development, we suggest that *ATXN10* haploinsufficiency may also participate in the neurodevelopment of PMS patients.

The *CELSR1* gene is highly likely to be LoF intolerant (pLI = 1) and is frequently lost or disrupted in individuals with PMS [4]. Recent work has shown that *CELSR1* mutations are associated with severe neural tube defects in humans [5],and *Celsr1* plays important roles in WNT signaling pathways [6].In addition, *CELSR1* and *SHANK3* are pleotropic genes with mostly complementary functions [4].

The *WNT7B* gene is part of the WNT signaling pathway in mammals, and mouse *WNT7B* plays a role in the formation of the brain vasculature [7]. Manipulations of *WNT7B* can modify dendritic branching in the hippocampus [8]. Proteins encoded by the *WNT7B* gene were found to physically interact with proteins associated with ASDs, ID, hypotonia and macrocephaly [9]. *WNT7B* is very close to *CELSR1* (384 kb).

*FBLN1* (fibulin 1), which is most likely sensitive to LoF variants (pLI = 0.85) and disrupted in approximately half of all individuals with PMS [4]. *FBLN1* is associated with autosomal dominant syndactyly in humans (OMIM#680180). Haploinsufficiency of the *FBLN1*-D variant leads to the observed limb malformations [10].In addition, Bohlega et al. found that mutation of fibulin-1 involves the central nervous system and connective tissue [11]. Thus, more work is needed to determine whether this loss may also contribute to developmental defects of the brain and/or vasculature.

References

1. Waragai M, Nagamitsu S, Xu W, Li YJ, Lin X, Ashizawa T. Ataxin 10 induces neuritogenesis via interaction with G-protein beta2 subunit.J Neurosci Res 2006; 83: 1170-1178.

2. Matsuura T, Yamagata T, Burgess DL, Rasmussen A, Grewal RP, Watase K, et al. Large expansion of the ATTCT pentanucleotide repeat in spinocerebellar ataxia type 10.Nat Genet 2000; 26: 191-194.

3. Keren B, Jacquette A, Depienne C, Leite P, Durr A, Carpentier W, et al. Evidence against haploinsuffiency of human ataxin 10 as a cause of spinocerebellar ataxia type 10.Neurogenetics 2010; 11: 273-274.

4. Mitz AR, Philyaw TJ, Boccuto L, Shcheglovitov A, Sarasua SM, Kaufmann WE, et al. Identification of 22q13 genes most likely to contribute to Phelan McDermid syndrome.Eur J Hum Genet 2018; 26: 293.

5. Allache R, De Marco P, Merello E, Capra V, Kibar Z. Role of the planar cell polarity gene *CELSR1* in neural tube defects and caudal agenesis.Birth Defects Res A Clin Mol Teratol 2012; 94: 176-181.

6. Yang Y, Mlodzik M. Wnt-Frizzled/planar cell polarity signaling: cellular orientation by facing the wind (Wnt).Annu Rev Cell Dev Biol 2015; 31: 623-646.

7. Stenman JM, Rajagopal J, Carroll TJ, Ishibashi M, McMahon J, McMahon AP. Canonical Wnt signaling regulates organ-specific assembly and differentiation of CNS vasculature.Science 2008; 322: 1247-1250.

8. Rosso SB, Sussman D, Wynshaw-Boris A, Salinas PC. Wnt signaling through Dishevelled, Rac and JNK regulates dendritic development.Nat Neurosci 2005; 8: 34-42.

9. Sarasua SM, Dwivedi A, Boccuto L, Chen C-F, Sharp JL, Rollins JD, et al. 22q13.2q13.32 genomic regions associated with severity of speech delay, developmental delay, and physical features in Phelan–McDermid syndrome.Genet Med 2014; 16: 318-328.

10. Debeer P, Schoenmakers EF, Twal WO, Argraves WS, De Smet L, Fryns JP, et al. The fibulin-1 gene (*FBLN1*) is disrupted in a t(12;22) associated with a complex type of synpolydactyly.J Med Genet 2002; 39: 98-104.

11. Bohlega S, Al-Ajlan H, Al-Saif A. Mutation of fibulin-1 causes a novel syndrome involving the central nervous system and connective tissues.Eur J Hum Genet 2014; 22: 640-643.
